# Altitude conditions seem to determine the evolution of COVID-19 in Brazil

**DOI:** 10.1038/s41598-021-83971-x

**Published:** 2021-02-23

**Authors:** José Sebastião Cunha Fernandes, Ricardo Siqueira da Silva, Alexandre Christófaro Silva, Daniel Campos Villela, Vanessa Amaral Mendonça, Ana Cristina Rodrigues Lacerda

**Affiliations:** 1 Faculdade de Ciências Agrárias, Universidade Federal dos Vales do Jequitinhonha e Mucuri (UFVJM), Diamantina, Minas Gerais Brazil; 2 Centro Integrado de Pós-Graduação e Pesquisa em Saúde (CIPq-Saúde), Universidade Federal dos Vales do Jequitinhonha e Mucuri (UFVJM), Diamantina, Minas Gerais Brazil

**Keywords:** Environmental sciences, Environmental social sciences, Diseases, Health care, Medical research

## Abstract

COVID-19 is spreading rapidly in Brazil, a country of continental dimensions, but the incidence of the disease is showing to be very heterogeneous, affecting cities and regions differently. Thus, there is a gap regarding what factors would contribute to accentuate the differences in the incidence of COVID-19 among Brazilian cities. This work aimed to evaluate the effect of altitude on the incidence of COVID-19 in Brazilian cities. We analyzed the relative incidence (RI), the relative death rate (RDR) of COVID-19, and air relative humidity (RH) in all 154 cities in Brazil with a population above 200 thousand inhabitants, located between 5 and 1135 m in altitude. Pearson's correlation analysis was performed to compare a relationship between altitude with RI and RDR, and between RH with RI and RDR. Altitudes were classified into three classes [low (altitude ≤ 97 m a. s. l), middle (97 < altitude ≤ 795 m a. s. l), high (795 < altitude ≤ 1135 m a. s. l)] for the RI, RDR, and RH variables. To compare the three classes of altitude, analysis of variance (ANOVA) and Tukey test were used to compare averages (p < 0.05). Our epidemiological analysis found that the RI, RDR, and RH were lower in cities located in high altitudes (795 < altitude ≤ 1135 m a. s. l) when compared to the middle (97 < altitude ≤ 795 m a. s. l) and low (altitude ≤ 97 m a. s. l) cities altitudes. Furthermore, our study shows that there is a negative correlation between the incidence of COVID-19 with altitude and a positive correlation with RH in the cities analyzed. Brazilian cities with high altitude and low RH have lower RI and RDR from COVID-19. Thus, high altitude cities may be favorable to shelter people at risk. This study may be useful for understanding the behavior of SARS-CoV2, and start point for future studies to establish causality of environmental conditions with SARS-CoV2 contributing to the implementation of measures to prevent and control the spread of COVID-19.

## Introduction

In December 2019, a new coronavirus was identified—initially called 2019-nCoV and later renamed SARS-CoV-2—in Hubei province, People's Republic of China^1^. The pathogen causes coronavirus-19 disease (COVID-19), which spread rapidly, reaching the pandemic level on March 11, 2020^2^. The incidence of cases and deaths caused by COVID-19 in the world increased at different rates from the first cases. In Brazil, a country of continental dimensions, the spread of the disease is very heterogeneous, affecting cities and regions differently.

Efforts to minimize its spread are announced every minute in the media. Also, thousands of searches worldwide are focused on the various nuances of COVID-19. However, studies that relate to the incidence of the disease to environmental factors are incipient, especially in countries like Brazil, which stands out for its great socioeconomic and environmental diversity^2^. As the behaviour of COVID-19 concerning climatic attributes is still poorly understood, investigating the influence of altitude and environmental characteristics of cities on the incidence and deaths caused by COVID-19 can generate results that contribute to the development of public policies that minimize the spread of the disease.

In Brazil, COVID-19 began to be disseminated from large cities located in the coastal zone, that have high air relative humidity (RH), and quickly reached the interior, but the contamination speed of the population is not homogeneous^2^. Some cities have higher incidence and deaths from COVID-19 than other cities with similar numbers of inhabitants. About that, some questions were raised about the differences between the records of infection and deaths between cities. What factors would be able to contribute to accentuate these differences in the incidence of COVID-19 among Brazilian cities? Thus, the hypothesis arose that cities located at higher altitudes are less favourable for the spread of COVID-19.

Since, cities outside the coastal zone with a high incidence of COVID-19, such as Manaus—Amazonas, are located at low altitudes (< 100 m a. s. l). Brazil has 154 cities with a population above 200 thousand inhabitants located at altitudes ranging from 5 to 1135 m a. s. l. The incidence of COVID-19 in these cities is not similar. Could altitude be a determining factor that would influence the incidence of this virus?

During the survey of epidemiological data from Brazil, in May 2020, a study was published showing the relationship between altitude and the spread of COVID-19 based on epidemiological data from China, Bolivia, and Ecuador^3^. The authors showed that inhabitants are less susceptible to develop serious adverse effects caused by COVID-19 in cities with altitudes above 3000 m a.s.l.^3^. These authors related this low incidence to compensatory adjustments in physiological systems and environmental factors, such as higher ultraviolet radiation and thinner atmosphere. A recent review examined geographic components (altitude) and physiological factors associated with high altitude in regards to the incidence and severity of COVID-19 infections. There were found clues that physiological and genetic aspects of high altitude populations may influence the pattern of SARS-CoV-2 expansion and COVID-19 infection severity^4^. Thus, altitude can be a determining factor in the transmission rate of COVID-19. However, due to the importance and urgency of understanding the disease and its behaviour in different locations, it is necessary to highlight the relationships of COVID-19 in other countries, especially in those with greater altitudinal and environmental diversity, such as Brazil.

This work aims to evaluate the incidence of COVID-19 in 154 Brazilian cities with a population above 200 thousand inhabitants, located between 5 and 1135 m in altitude. The results may be useful to apply public policies for the prevention and control of the spread of COVID-19 in Brazil and the world.

## Material and methods

### Cities data

**Figure 1:**
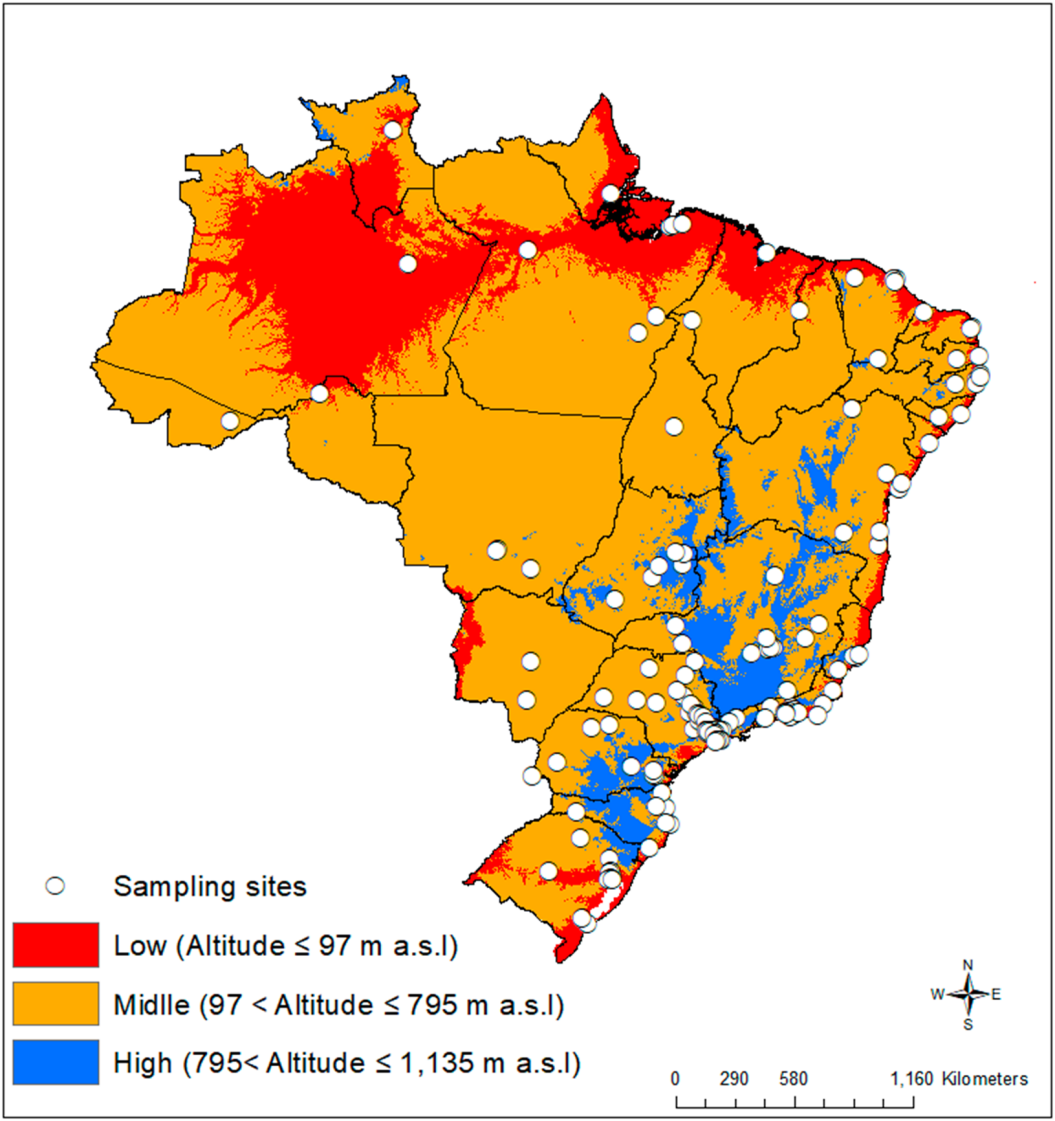
Distribution of 154 Brazilian cities with a population above 200 thousand inhabitants selected to analyse the relative incidence and relative deaths per 100.000 inhabitants of Coronavirus Disease-19 (COVID-19) cases according to three classes of altitudes. Low (red), middle (orange) and high (blue). The information is available in free spatial data DIVA-GIS (http://www.diva-gis.org/Data). The map was made using Quantum geographic information system (QGIS) version 3.14.15^6^.

Data from all 154 Brazilian cities with a population above 200 thousand inhabitants (Fig. 1) were collected on May, June, and July, 2020. These cities are located between 5 and 1135 m in altitude, have 98,080,747 inhabitants (46.7% of the Brazilian population—Brazilian Institute of Geography and Statistics—IBGE)^5^. Figure 1 shows the sampled locations, and the different altitudes in Brazil divided into three classes based on the available data DIVA-GIS (http://www.diva-gis.org/Data).

### Data collect

The altitude data for each city was carefully obtained from the average of five measurements obtained in their respective north, south, east, west, and central positions, from Google Earth at https://earth.google.com/web/@0,0,-24018.82718741a,36750128.22569847d,35y,0h,0t,0r/data=KAE (Google Earth, 2020).

The population for each city was obtained using data from IBGE, referring to July 1, 2019, at https://agenciadenoticias.ibge.gov.br/agencia-detalhe-de-midia.html?view=mediaibge&catid=2103&id=3098^5^.

Data on confirmed cases and deaths, resulting from COVID-19, were obtained, on May 17, 2020, on June 01 and 16, 2020 and on July 01, 2020 at https://covid.mapbiomas.org/^7^.

The data regarding RH were obtained from the National Institute of Meteorology address http://www.inmet.gov.br/portal/index.php?r=estacoes/estacoesAutomaticas^8^, for the 63, 58, 57 and 59 cities with a population over 200 thousand inhabitants and that have data from automatic weather stations and correspond respectively to the average of the period from March 01 to May 17, May 18 to June 01, 2020, June 02 to June 16, 2020 and June 17 to July 01, 2020.

### Statistical analysis

The COVID-19 incidence and death data were calculated per 100,000 inhabitants and are called relative incidence (RI) and relative death rate (RDR). This objective procedure removes the effect of the number of inhabitants in the analyses.

Pearson's correlation analysis was performed to compare a relationship between altitude with RI and RDR, and between RH with RI and RDR.

Define as a predetermined variable as altitudes were divided into three classes [low (altitude ≤ 97 m a. s. l) with 65 cities, middle (97 < altitude ≤ 795 m a. s. l) with 66 cities, high (795 < altitude ≤ 1135 m a. s. l with 23 cities)] for the RI, RDR variables and the same classes for the RH variable. However, for the RH were considered 63 cities (March 01 to May 17, 2020), 58 cities (May 18 to June 01, 2020), 57 cities (June 02 to June 16, 2020), and 56 cities (June 17 to July 01, 2020) according to data provided by the National Institute of Meteorology. We verified that high altitude cities had lower COVID-19 RI and RDR than cities of low altitude. We defined three classes to test our hypotheses, assuming that there would be a staggered decrease in RI and RDR with increasing altitude. To compare three classes of altitude (until May 17 and until June 1, 2020 for the variables RI and RDR and between March 1 and May 17 and between May 18 and June 1 for the RH variable), analysis of variance (ANOVA) and Tukey test were used to compare media (p < 0.05). The COVID-19 mortality and death data were transformed into a log (X + 1) to stabilize the variance, and the non-transformed data were submitted to ANOVA. Both show significance. Thus, we prefer to display a statistical output of unprocessed data as analyses and figures were performed in Excel and Sigma plot 12.5 software^9^.

### Consent for publication

The researchers of this study confirm that they have given due consideration to protect the intellectual property associated with this work and that there are no impediments to publication, including the timing of publication, with respect to intellectual property. In so doing we confirm that we have followed the regulations of our institutions concerning intellectual property.

## Results

The 154 municipalities studied correspond to 2.76% of the Brazilian municipalities (5570 municipalities) but are home to 46.7% of the Brazilian population (98,080,747 inhabitants) (Table 1). A total of 13 municipalities are in the central west region with 53.4% of the region's population; 12 are in the northern region with 39.59% of its population; 74 are in the south-eastern region and with 58.7% of the region's population; 28 are in the northeast region with 32.99% of the region's population, and 27 are located in the south region with 38% of the region's population.

**Table 1:** Cities and regions of Brazil, altitude, population, relative incidence (RI), relative death rate (RDR), air relative humidity (RH). Data from the first case, and acceleration of the incidence of COVID-19.

City	Region	Altitude (m)	Population (no.)	RI1 (100,000 hb-1)	RDR1 (100,000 hb-1)	RH3 (%)	1º cases (data)	IA (data)	RH4 (%)	RI2 (100,000 hb-1)	RDR2 (100,00 0 hb-1)	RH5 (%)	RI6 (100,000 hb-1)	RDR6 (100,00 hb-1)	RH7 (%)	RI8 (100.000 hb-1)	RDR8 (100.000 hb-1)
Cuiabá	CW	195	612,547	44.73	0.33	63.6	20/Mar	31/Mar	62.5	129.79	1.80	62	316.87	10.94	–	647.13	30.37
Várzea Grande	CW	195	284,971	26.67	1.40	–	23/Mar	08/May	–	90.18	4.21	–	209.50	17.55	–	463.91	44.57
Rondonópolis	CW	260	232,491	39.14	1.29	72.1	30/Mar	13/Apr	69.2	88.61	3.01	66.7	245.17	8.17	61.7	522.60	20.65
Palmas	CW	266	299,127	83.24	1.00	75.2	18/Mar	28/Apr	69.0	204.26	2.67	58.4	349.35	4.01	51.2	629.83	6.35
Dourados	CW	445	222,949	18.84	0.45	52	28/Mar	15/May	–	125.14	0.90	59.4	579.50	2.24	55.6	1,197.58	10.76
Campo Grande	CW	590	895,982	19.42	0.56	60.4	14/Mar	15/Apr	60.6	32.70	0.78	73.5	87.50	0.89	61.9	278.02	1.12
Rio Verde	CW	740	235,647	10.61	0.85	56.4	12/Mar	29/Apr	62.0	46.26	0.85	–	782.10	2.12	–	1,785.30	14.85
Goiânia	CW	787	1,516,113	60.75	1.98	72.5	12/Mar	26/Mar	68.2	114.11	3.50	62.3	253.87	6.27	60.9	458.80	11.02
Aparecida de Goiânia	CW	803	578,179	26.81	1.21	–	18/Mar	27/Apr	–	78.18	1.90	–	195.10	3.98	–	464.39	9.17
Luziânia	CW	980	208,299	20.64	1.44	74.9	25/Mar	14/May	70.3	39.37	1.92	63	160.35	2.88	62.1	345.18	3.36
Anápolis	CW	1043	386,923	13.44	0.52	–	16/Mar	12/Apr	–	40.83	0.52	–	108.29	1.29	–	223.04	1.29
Brasília	CW	1,075	3,015,268	112.49	1.79	78.4	07/Mar	24/Mar	72.2	292.08	5.21	67.7	705.84	9.55	65.2	1452.61	17.88
Águas Lindas de Goiás	CW	1,135	212,440	13.18	0.47	–	28/Mar	29/Apr	–	98.85	2.35	–	193.00	6.59	–	345.04	11.77
Region mean		654.9	669,303														
Aracaju	NE	9	657,013	309.28	3.04	–	14/Mar	28/Apr	–	618.10	10.81	–	1499.06	24.35	–	2204.83	41.40
Jaboatão dos Guararapes	NE	10	702,298	266.84	24.21	–	17/Mar	07/Apr	–	454.22	45.28	–	570.27	59.52	–	687.88	80.73
Fortaleza	NE	13	2,669,342	575.83	43.98	80.2	16/Mar	18/Mar	79.9	902.99	78.07	79.4	1160.81	112.61	73.2	1322.91	123.18
Recife	NE	17	1,645,727	593.78	36.21	79.5	12/Mar	28/Mar	84.8	959.09	65.69	84.3	1113.98	93.45	79.5	1259.56	110.89
Cabo de Santo Agostinho	NE	23	207,048	171.46	26.08	–	03/Apr	26/Apr	–	312.97	45.88	–	393.15	59.41	–	445.31	77.76
João Pessoa	NE	23	809,015	209.76	9.27	78.5	18/Mar	02/Apr	82.6	489.36	16.07	81.5	1046.21	28.80	76.4	1569.07	39.31
Paulista	NE	23	331,774	354.76	22.00	–	03/Apr	09/Apr	–	568.16	44.91	–	702.59	53.05	–	793.91	60.88
Natal	NE	25	884,122	134.03	3.96	82.4	12/Mar	28/Mar	87.3	291.36	9.61	84.2	609.31	21.38	82.7	1312.38	49.99
Olinda	NE	27	392,482	419.38	26.75	–	22/Mar	09/Apr	–	667.04	43.31	–	799.78	51.72	–	963.36	57.33
Caucaia	NE	31	361,400	245.71	10.51	–	28/Mar	13/Apr	–	499.17	24.90	–	848.92	65.58	–	996.40	77.20
Mossoró	NE	32	297,378	168.81	7.73	73.7	21/Mar	03/Apr	–	331.56	16.14	–	555.52	28.92	–	959.39	42.37
São Luís	NE	32	1,101,884	529.09	35.76	89.4	20/Mar	30/Mar	86.9	839.93	47.74	85.8	1097.67	59.35	84.9	1192.87	75.69
Salvador	NE	36	2,872,347	185.56	6.58	83.6	13/Mar	21/Mar	84.9	397.97	16.50	84.7	736.75	26.98	81.4	1166.47	40.49
Maracanaú	NE	41	227,886	230.82	27.21	–	29/Mar	05/Apr	–	716.15	50.90	–	1231.76	82.50	–	1587.64	93.47
Camaçari	NE	44	299,132	31.76	1.34	–	20/Mar	23/Apr	–	69.53	1.34	–	195.23	4.35	–	463.34	7.69
Parnamirim	NE	44	261,469	84.14	2.29	–	20/Mar	16/Apr	–	211.11	3.82	–	526.26	13.00	–	1116.00	29.83
Itabuna	NE	61	213,223	298.75	5.16	–	19/Mar	07/Apr	–	419.28	15.48	–	714.28	19.70	–	1210.00	26.26
Maceió	NE	65	1,018,948	156.34	13.25	79.1	08/Mar	13/Apr	–	598.36	28.26	–	1108.40	47.40	–	1511.76	58.88
Sobral	NE	72	208,935	261.8	9.09	–	18/Mar	22/Apr	–	1063.49	34.94	–	2042.74	84.24	–	3142.13	109.12
Teresina	NE	115	864,845	145.69	4.51	78.9	19/Mar	30/Mar	67.0	266.41	10.41	62	563.92	25.67	–	959.02	44.75
Feira de Santana	NE	235	614,872	33.67	0.33	80.8	06/Mar	30/Mar	87.5	79.04	0.98	88.1	177.27	4.55	85.9	519.78	7.81
Arapiraca	NE	263	231,747	56.1	2.59	–	18/Apr	27/Apr	–	180.37	5.18	–	647.26	10.79	–	1296.24	15.53
Juazeiro	NE	370	216,707	12	0.92	–	23/Mar	14/May	–	18.92	2.31	–	83.52	3.69	–	286.56	8.77
Petrolina	NE	380	349,145	24.63	1.15	63.2	23/Mar	06/May	64.2	44.68	2.29	65.2	69.60	3.44	63.7	195.05	7.16
Juazeiro do Norte	NE	408	274,207	17.87	2.55	–	20/Mar	26/Apr	–	102.84	4.01	–	312.17	17.14	–	625.44	32.09
Campina Grande	NE	512	409,731	70.78	1.22	80.9	27/Mar	18/Apr	87.7	370.73	4.88	86.2	1101.21	14.89	85.1	1587.63	25.14
Caruaru	NE	565	361,118	38.49	3.32	85.5	23/Mar	18/Apr	91.4	104.95	9.14	91.9	230.40	16.06	–	481.01	32.68
Vitória da Conquista	NE	970	341,597	19.61	1.17	–	01/Apr	12/Apr	–	36.89	1.46	–	113.29	1.46	–	209.90	4.39
Region mean		158.79	672,335														
Macapá	N	11	503,327	531.26	14.3	84.6	20/Mar	03/Apr	82.3	1009.09	27.02	82	1508.56	40.33	82.2	2581.82	50.86
Belém	N	20	1,492,745	391.43	43.68	69.4	18/Mar	06/Apr	82.5	796.18	89.43	81.2	1103.81	119.11	78.5	1314.42	128.62
Ananindeua	N	21	530,598	231.63	20.73	–	25/Mar	15/Apr	–	519.98	45.42	–	690.73	57.67	–	824.54	62.57
Castanhal	N	39	200,793	158.37	22.41	87.1	26/Mar	19/Apr	83.8	357.58	47.31	81.6	642.45	72.21	80.8	794.35	82.67
Santarém	N	44	304,589	84.7	5.91	–	01/Apr	30/Apr	–	332.58	30.86	–	540.07	48.92	–	1093.28	56.47
Manaus	N	60	2,182,763	476.78	43.48	79.7	13/Mar	22/Mar	79.9	838.07	62.44	78.1	1081.75	75.36	73.8	1244.71	82.01
Boa Vista	N	80	399,213	314.12	8.27	–	23/Mar	13/Apr	–	655.04	24.05	78.6	1304.32	41.08	79.3	2751.91	56.86
Porto Velho	N	86	529,544	284.96	9.63	–	21/Mar	14/Apr	–	660.00	20.58	–	1536.42	46.27	–	2480.06	68.93
Marabá	N	97	279,349	71.95	8.95	82.9	23/Mar	27/Apr	82.4	140.33	33.65	76.8	824.77	39.74	69.1	1426.89	49.76
Imperatriz	N	146	258,682	329.36	13.92	78.1	01/Apr	17/Apr	73.2	786.29	38.66	66.2	1252.89	62.63	61.0	1570.27	78.47
Rio Branco	N	157	407,319	324.81	13.01	85.2	17/Mar	17/Apr	–	1007.56	28.48	–	1339.74	48.12	–	1719.05	64.57
Parauapebas	N	180	208,273	90.27	11.04	–	28/Mar	24/Apr	–	751.90	29.77	–	2998.95	47.05	–	4758.18	57.62
Region mean		78.42	608,100														
Guarujá	SE	9	320,459	115.46	4.68	–	31/Mar	14/Apr	–	312.05	16.23	–	731.45	28.08	–	1152.72	40.57
Campos dos Goytacazes	SE	10	507,548	36.84	1.97	77.6	24/Mar	27/Apr	76.4	146.00	6.11	79.2	239.39	11.62	75.3	353.86	17.93
Angra dos Reis	SE	14	203,785	179.11	7.36	83.8	30/Mar	28/Apr	–	437.23	16.68	–	732.14	28.46	–	1077.12	40.73
Macaé	SE	14	256,672	43.64	6.23	71.9	31/Mar	16/Apr	79.5	326.10	10.91	84.2	617.13	21.04	74.2	923.75	30.00
Santos	SE	14	433,311	322.4	19.85	–	30/Mar	01/Apr	–	802.66	34.16	–	1454.15	51.93	–	2140.73	85.39
Magé	SE	15	245,071	142	13.87	–	02/Apr	23/Apr	–	267.27	36.32	–	493.33	45.70	–	661.03	53.86
Cabo Frio	SE	17	219,863	60.95	2.73	–	07/Apr	23/Apr	–	171.47	8.19	–	256.07	11.83	–	396.61	19.56
Vitória	SE	17	362,097	344.94	15.47	70.0	19/Mar	05/Apr	69.4	677.44	26.51	65.6	1406.25	49.99	66.1	2023.21	69.59
São Gonçalo	SE	18	1,084,839	58.26	5.99	–	23/Mar	07/Apr	–	149.24	14.66	–	271.56	25.72	–	470.39	39.64
Vila Velha	SE	18	493,838	277.82	9.72	81.6	19/Mar	08/Apr	81	490.65	20.05	82.6	1012.07	38.88	81.5	1550.51	56.90
Niterói	SE	20	513,584	231.32	12.66	76.5	12/Mar	26/Mar	73.7	596.79	20.25	79.4	909.49	28.43	68.3	1241.67	44.78
Duque de Caxias	SE	22	919,596	99.94	15.77	74.1	24/Mar	07/Apr	56.5	163.33	27.19	–	241.19	37.08	–	338.19	46.76
Rio de Janeiro	SE	23	6,718,903	192.31	27.4	78.3	06/Mar	18/Mar	69.4	433.95	53.25	80.2	630.83	75.76	68.1	847.40	97.49
São Vicente	SE	23	365,798	112.36	4.1	–	31/Mar	15/Apr	–	268.73	21.87	–	454.08	30.34	–	836.53	45.11
Praia Grande	SE	24	325,073	89.83	13.23	–	01/Apr	15/Apr	–	215.95	17.23	–	582.02	20.92	–	777.98	26.46
Itaboraí	SE	25	240,592	152.54	13.3	–	27/Mar	04/Apr	–	470.09	32.42	–	728.62	44.47	–	1023.31	51.96
Nova Iguaçu	SE	27	821,128	93.29	11.57	–	28/Mar	04/Apr	–	142.49	22.16	–	314.08	33.98	–	397.75	41.65
Belford Roxo	SE	32	510,906	73.01	9.98	–	26/Mar	11/Apr	–	139.16	19.77	–	214.13	29.16	–	279.50	34.64
São João de Meriti	SE	32	472,406	99.07	10.16	–	30/Mar	09/Apr	–	183.74	18.20	–	279.63	32.39	–	350.33	45.51
Cachoeiro de Itapemirim	SE	62	208,972	38.28	1.44	–	20/Mar	29/Apr	–	126.33	5.74	–	449.34	13.88	–	798.67	29.19
Cariacica	SE	62	381,285	237.09	9.44	–	20/Mar	13/Apr	–	480.22	24.13	–	935.78	46.42	–	1344.93	63.21
Serra	SE	65	517,510	257.58	15.07	–	23/Mar	01/Apr	–	484.63	30.34	–	984.72	46.57	–	1481.71	59.71
Governador Valadares	SE	165	279,885	25.72	1.79	80.0	26/Mar	25/Apr	80.2	67.17	2.50	80.6	148.28	3.57	80.1	406.24	12.86
Ipatinga	SE	245	263,410	13.67	0	–	12/Mar	15/Apr	–	93.39	0.38	–	268.78	6.45	–	750.54	15.94
Volta Redonda	SE	400	273,012	163.73	6.59	–	24/Mar	02/Apr	–	254.93	13.19	–	413.17	18.68	–	619.75	20.88
Presidente Prudente	SE	430	228,743	39.35	2.19	58.0	08/Apr	24/Apr	55.2	60.33	2.62	69.9	105.36	5.25	59.5	291.59	8.31
São José do Rio Preto	SE	518	460,671	77.93	2.6	–	18/Mar	07/Apr	–	151.95	4.99	–	282.41	8.47	–	563.74	17.37
Bauru	SE	559	376,818	58.12	3.45	69.3	31/Mar	15/Apr	65.2	78.02	3.98	77.6	129.77	4.51	69.8	363.31	6.90
Piracicaba	SE	570	404,142	55.92	4.21	64.6	30/Mar	27/Apr	50.4	141.53	7.18	63.2	277.63	10.89	50.3	508.73	21.77
Ribeirão Preto	SE	570	703,293	63.98	1.85	–	26/Mar	04/Apr	–	134.08	3.41	–	281.25	9.10	–	562.64	22.18
Limeira	SE	580	306,114	23.85	0.33	–	01/Apr	28/Apr	–	50.96	1.31	–	118.26	6.86	–	266.24	11.76
Americana	SE	582	239,597	27.55	1.67	–	31/Mar	18/Apr	–	50.50	2.50	–	101.84	4.59	–	225.80	10.02
Jacareí	SE	590	233,662	37.23	1.71	–	08/Apr	17/Apr	–	92.44	3.85	–	166.48	8.99	–	292.30	12.41
Rio Claro	SE	590	206,424	12.6	4.36	–	25/Mar	17/Apr	–	23.25	5.33	–	111.91	9.20	–	300.35	14.53
Sumaré	SE	590	282,441	41.07	1.06	–	07/Apr	06/May	–	68.69	2.48	–	145.52	4.60	–	383.80	11.33
Taubaté	SE	595	314,924	18.73	1.27	74.1	19/Mar	27/Apr	70.4	43.82	1.59	77.9	60.65	1.59	73.9	153.05	2.54
Sorocaba	SE	600	679,378	107.45	4.27	72.5	27/Mar	25/Apr	63.5	135.42	6.48	76.3	230.80	10.89	69.5	632.93	17.96
São José dos Campos	SE	610	721,944	62.33	2.77	–	18/Mar	04/Apr	–	124.80	4.85	–	212.34	7.76	–	407.65	12.74
Indaiatuba	SE	615	251,627	22.65	3.97	–	01/Apr	17/Apr	–	58.02	7.95	–	79.88	10.33	–	233.68	17.49
Hortolândia	SE	620	230,851	45.92	5.63	–	20/Mar	18/Apr	–	66.71	5.20	–	134.29	8.66	–	334.41	11.26
Marília	SE	640	238,882	13.81	0.42	65.9	03/Apr	15/May	57.5	25.95	0.42	73.7	66.14	1.67	65.0	149.86	5.02
Montes Claros	SE	660	409,341	9.77	0.49	72.4	06/Apr	08/May	68.2	16.86	0.49	62.5	34.69	0.73	60.1	64.98	0.98
Campinas	SE	682	1,204,073	81.97	3.07	–	18/Mar	07/Apr	–	151.32	6.48	–	322.57	12.37	–	666.07	26.24
Araraquara	SE	694	236,072	59.73	1.69	–	02/Apr	15/Apr	–	99.12	1.69	–	240.60	2.54	–	402.84	4.66
Divinópolis	SE	745	238,230	53.31	0.42	80.6	08/Mar	01/Apr	78.7	79.34	0.84	76.7	106.20	1.26	75.9	138.52	4.62
Guarulhos	SE	750	1,379,182	110.79	11.96	–	17/Mar	01/Apr	–	200.77	20.59	–	307.72	34.01	–	496.16	49.45
Jundiaí	SE	754	418,962	95.47	6.92	–	25/Mar	16/Apr	–	245.13	13.61	–	482.86	26.26	–	865.71	42.49
Itaquaquecetuba	SE	760	370,821	92.77	9.17	–	31/Mar	15/Apr	–	160.45	19.42	–	241.90	27.24	–	381.05	35.87
Carapicuíba	SE	770	400,927	147.16	9.73	–	14/Mar	09/Apr	–	257.90	18.96	–	413.29	27.69	–	623.06	36.17
Mogi das Cruzes	SE	770	445,842	137.49	9.64	–	20/Mar	10/Apr	–	223.85	19.07	–	316.03	27.14	–	466.76	35.89
Sete Lagoas	SE	770	239,639	4.59	0.42	76.5	17/Mar	19/Apr	72.9	12.10	0.42	68	18.78	0.42	65.3	95.14	1.67
Juiz de Fora	SE	777	568,873	72.42	2.81	73.5	14/Mar	31/Mar	76.7	108.46	5.27	79.5	154.87	6.68	77.3	296.20	9.32
Diadema	SE	778	423,884	150.28	12.03	–	27/Mar	07/Apr	–	279.09	19.82	–	478.20	30.20	–	768.13	41.28
Suzano	SE	780	297,637	125.32	12.77	–	19/Mar	11/Apr	–	222.08	16.80	–	354.12	28.89	–	493.22	34.94
Itapevi	SE	781	237,700	137.99	14.72	–	30/Mar	15/Apr	–	225.07	26.92	–	335.72	42.49	–	490.95	55.95
Osasco	SE	785	698,418	247.85	29.07	–	18/Mar	31/Mar	–	389.17	44.53	–	592.77	57.27	–	832.17	69.30
São Paulo	SE	785	12,252,023	293.27	23.14	81.3	25/fev	11/Mar	73.4	498.91	35.13	83.5	762.71	46.13	74.3	1037.53	58.46
Barueri	SE	786	274,182	265.52	29.91	76.8	18/Mar	16/Apr	68.5	433.29	46.32	78.7	599.97	62.00	70.9	767.37	73.67
Santo André	SE	790	718,773	180.31	9.46	–	16/Mar	30/Mar	–	322.49	18.50	–	552.47	27.41	–	889.02	35.62
Taboão da Serra	SE	790	289,664	136.02	10.36	–	25/Mar	01/Apr	–	283.78	22.44	–	475.38	34.87	–	733.95	44.53
Uberaba	SE	790	333,783	29.96	1.50	75.7	20/Mar	06/Apr	74.5	59.92	1.80	70.2	115.04	4.19	72.1	221.70	7.79
São Bernardo do Campo	SE	795	838,936	172.36	14.3	–	16/Mar	30/Mar	–	313.73	25.27	–	554.51	34.09	–	923.43	44.46
Santa Luzia	SE	800	219,134	9.13	0.46	–	29/Mar	13/May	–	21.90	0.46	–	65.26	0.91	–	145.12	2.74
Mauá	SE	805	472,912	122.01	8.04	–	16/Mar	07/Apr	–	217.17	12.48	–	350.17	23.89	–	481.48	30.87
Embu das Artes	SE	810	273,726	117.64	9.86	–	25/Mar	02/Apr	–	202.39	16.81	–	313.09	25.21	–	450.09	32.15
Cotia	SE	820	249,210	139.64	13.64	–	18/Mar	31/Mar	–	239.96	20.06	–	355.52	26.48	–	564.99	35.71
Betim	SE	830	439,340	8.65	0.68	–	23/Mar	06/May	–	28.45	1.37	–	90.59	3.87	–	190.29	7.28
Ribeirão das Neves	SE	870	334,858	6.87	0.3	–	02/Apr	27/Apr	–	24.19	0.60	–	58.23	0.90	–	168.43	2.99
São Carlos	SE	870	251,983	22.62	1.19	68.0	06/Apr	09/May	64.9	48.81	1.98	72.7	100.80	2.78	68.9	194.46	5.16
Petrópolis	SE	889	306,191	69.56	8.82	–	21/Mar	14/Apr	–	136.84	13.72	–	223.72	20.90	–	265.19	30.05
Uberlândia	SE	892	691,305	56.27	1.59	64.2	17/Mar	02/Apr	63.3	130.04	2.60	56.1	314.77	5.93	58.3	930.85	11.72
Belo Horizonte	SE	904	2,512,070	46.18	1.23	72.9	16/Mar	23/Mar	75.2	74.92	1.95	71	138.85	3.03	74.3	235.46	5.73
Contagem	SE	953	663,855	16.87	0.45	–	23/Mar	17/Apr	–	39.32	1.51	–	101.23	3.16	–	134.82	6.48
Franca	SE	962	353,187	10.19	0.57	66.4	02/Apr	28/Apr	62.1	23.22	0.57	–	28.88	1.70	60.2	68.24	2.55
**Region Mean**		**500.4**	**700,862**														
Rio Grande	S	5	211,005	4.74	0.47	–	23/Mar	02/May	–	9.00	0.47	–	32.70	0.95	–	94.31	1.90
Itajaí	S	6	219,536	80.17	1.37	80.0	21/Mar	27/Mar	75.6	209.99	4.56	86.3	460.06	9.11	83.9	828.11	15.94
Pelotas	S	12	342,405	10.22	0	–	25/Mar	14/Apr	–	25.12	0.00	–	47.90	0.00	–	81.19	0.88
Canoas	S	13	346,616	13.85	0.87	–	21/Mar	17/Apr	–	29.72	1.73	–	69.24	2.02	–	170.51	5.77
Florianópolis	S	22	500,973	94.42	1.2	70.6	12/Mar	23/Mar	71.1	137.13	1.40	77.7	191.63	1.80	73.5	287.04	2.79
Gravataí	S	32	281,519	10.66	0.36	–	25/Mar	02/Apr	–	22.38	1.42	–	52.93	1.78	–	142.09	3.91
Joinville	S	32	590,466	52.16	1.86	–	13/Mar	30/Mar	–	71.64	3.90	–	104.66	4.06	–	304.17	6.60
Novo Hamburgo	S	37	246,748	29.99	0.81	–	29/Mar	03/Apr	–	60.39	1.62	–	112.26	3.24	–	310.03	10.13
São Leopoldo	S	42	236,835	48.13	0.42	–	21/Mar	09/Apr	–	97.54	0.84	–	156.23	2.53	–	369.46	7.18
São José	S	44	246,586	36.09	0.41	–	19/Mar	09/Apr	–	50.29	0.81	–	101.38	0.81	–	185.74	3.65
Blumenau	S	47	357,199	117.02	0.84	–	22/Mar	06/Apr	–	184.49	1.12	–	226.76	1.68	–	454.37	2.52
Criciúma	S	52	215,186	130.58	2.79	–	20/Mar	09/Apr	–	178.45	4.18	–	226.78	4.18	–	294.16	5.11
Alvorada	S	57	210,305	10.94	3.8	–	19/Mar	17/Apr	–	31.86	1.43	–	73.23	1.90	–	184.02	4.28
Porto Alegre	S	58	1,483,771	40.57	1.42	71.7	11/Mar	20/Mar	76.3	50.14	2.49	83.0	105.68	3.84	76.8	181.97	6.20
Viamão	S	92	255,224	12.93	0.39	–	23/Mar	13/Apr	–	18.42	1.57	–	29.78	3.53	–	68.57	5.49
Santa Maria	S	116	282,123	15.6	0.71	68.9	22/Mar	08/Apr	78.9	65.57	1.06	86.2	151.71	2.84	90.7	205.23	6.03
Foz do Iguaçu	S	202	258,532	29.01	0.77	63.3	18/Mar	29/Mar	66.3	48.74	0.77	71.0	70.78	1.55	58.9	153.56	3.09
Maringá	S	518	423,666	21.72	1.42	56.3	18/Mar	21/Apr	57.7	38.71	1.42	74.1	106.69	2.60	65.4	274.27	4.25
Londrina	S	554	569,733	23.52	2.81	–	17/Mar	01/Apr	–	74.25	4.21	–	162.36	8.25	–	245.03	13.34
Passo Fundo	S	661	203,275	152.5	10.82	66.2	26/Mar	21/Apr	75.9	364.53	15.25	82.7	613.95	18.20	74.7	918.46	21.65
Chapecó	S	671	220,367	229.62	0	–	20/Mar	26/Apr	–	419.75	1.82	–	507.34	2.72	71.1	1129.48	4.99
Cascavel	S	743	328,454	42.02	1.52	–	23/Mar	03/Apr	–	140.05	2.13	–	337.34	5.48	–	861.00	14.01
Caxias do Sul	S	758	510,906	14.68	0.39	–	12/Mar	30/Mar	–	32.69	0.78	–	84.56	1.57	–	219.02	3.91
São José dos Pinhais	S	910	323,340	14.85	0.93	–	29/Mar	12/May	–	32.16	1.24	–	70.20	3.71	–	126.18	8.35
Ponta Grossa	S	930	351,736	9.1	0	–	21/Mar	23/Apr	–	20.47	0.00	–	42.36	0.28	–	99.22	0.28
Curitiba	S	940	1,933,105	33.06	1.71	71.6	12/Mar	19/Mar	59.8	50.90	2.43	69.1	98.18	4.29	66.0	212.87	7.50
Colombo	S	998	243,726	11.9	0	–	22/Mar	04/May	–	20.51	0.00	–	58.26	1.64	–	135.81	5.74
Region mean		316.7	421,975														
Total			98,080,747														

*IA* incidence acceleration, *RH* air relative humidity, *RI* relative incidence, *RDR* relative death rate, *CW* central-west, *NE* northeast, *N* north, *SE* southeast, *S* south. 1: until May 17, 2020. 2: until June, 01, 2020. 3: between March 01 and May, 17, 2020. 4: between May 18 and June, 01, 2020.5: 4: between June 02 and June, 16, 2020. 6: until June, 16, 2020; 7: between June 17 and July, 01, 2020; 8: until July, 01, 2020.

On May 17, 2020, these municipalities concentrated 71% of the cases (171,287) and 78% (12,610) of deaths due to COVID-19 in Brazil. On June, 01, 2020, these municipalities concentrated 63% of the cases (330,271) and 76% (22,567) of deaths due to COVID-19 in Brazil. On June, 16, 2020, these municipalities concentrated 59% of the cases (544,408) and 72% (32,829) of deaths due to COVID-19 in Brazil. On July, 01, 2020, these municipalities concentrated 56% of the cases (815,016) and 66% (42,949) of deaths due to COVID-19 in Brazil. The decrease in RDR in cities with a population over 200 thousand inhabitants, between May, 17 and July, 01, indicates that the COVID-19 virus is spreading to smaller cities.

The altitude varies between 5 and 1135 m a.s.l, and the cities located at higher altitudes are in the central-west region, followed by the southeast and south regions. The northern region has the lowest altitude gradient, and all cities are below 180 m a.s.l. (Table 1).

On May 17, 2020, the RI varied between 4.74 (Rio Grande) and 593.78 (Recife) (Table 1) and was higher in the north, followed by the northeast, southeast, central-west and, south (Table 2). On June 01, 2020, the RI varied between 12.10 (Sete Lagoas) and 1063.49 (Sobral) (Table 1) and was higher in the north, followed by the northeast, southeast, central-west and, south (Table 2). On June 16, 2020, the RI changed between 18.78 (Sete Lagoas) and 2998.95 (Parauapebas) (Table 1) and was higher in the north, followed by the northeast, southeast, central-west, and south (Table 2). On July 01, 2020, the RI changed between 64.98 (Montes Claros) and 4758.18 (Parauapebas) (Table 1) and was higher in the north, followed by the northeast, central-west, southeast, and south (Table 2).

**Table 2:** Mean relative incidence (RI), average relative death rate (RDR), average air relative humidity (RH) by geographic region and in Brazil.

Region	RI				RDR				RH (%)			
	May /17	June/01	June/16	July/01	May /17	June/01	June/16	July/01	May /17	June/01	June/16	July/01
NO	357.07	739.67	1163.09	1619.31	28.33	52.64	71.29	81.58	81.0	80.7	77.8	75.0
NE	284.35	529.16	837.25	1163.52	17.34	32.06	48.87	62.36	79.7	82.2	81.2	78.0
SE	156.84	297.06	483.79	719.44	13.5	22.45	35.59	44.87	73.3	69.1	74.3	69.1
SU	42.45	75.90	136.16	273.63	1.40	2.18	3.57	6.49	68.6	70.2	78.8	73.4
CO	60.46	160.78	407.94	884.84	1.34	3.23	6.86	14.31	67.3	66.8	64.1	59.0
Brazil mean	174.6	336.73	555.06	905.91	12.90	23.01	33.47	45.06	74.0	73.8	75.2	70.9

*CW* central-west, *NE* northeast, *N* north, *SE* southeast, *S* south.

**Figure 2:**
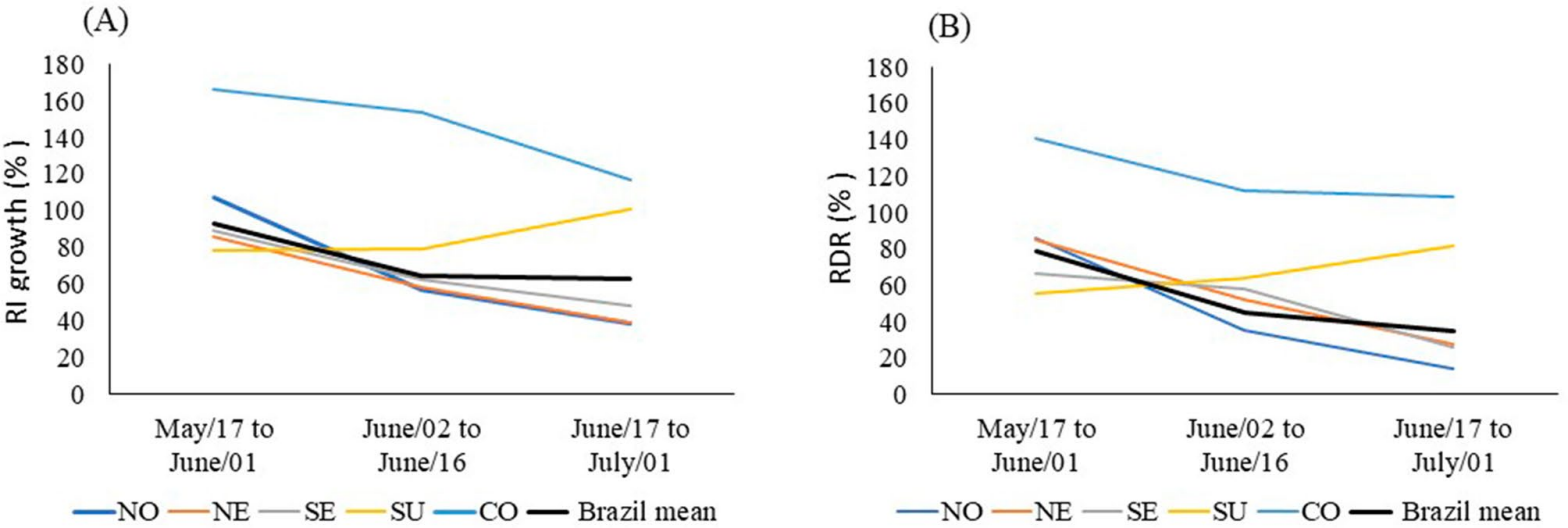
**(A)** Acceleration rate of relative incidence (RI), and **(B)** Acceleration rate of relative death rate (RDR) between May 17 and June 1, 2020; between June 2 and June 16, 2020; and between June 17 and July 01, 2020 in north (N), northeast (NE), southeast (SE), south (S), and central weast (CW) region.

In Brazil, between May 17 and June 01, between June 02 and June 16, and between June 17 and July, 01, 2020, the RI increased 92.3% 64.8%, 63.20%, respectively. The largest increase in RI was observed in the central-west region and the smallest in the northeast region (Table 2), and the rate of RI growth decelerated in all regions, except in the southern region (Fig. 2).

On May 17, 2020, the RDR varied between 0 (Chapecó, Colombo, Ipatinga, Pelotas and, Ponta Grossa) and 43.98 (Fortaleza) (Table 1) and was higher in the north, followed by the northeast, southeast, south and central-west (Table 2). On June 01, 2020, the RDR varied between 0 (Colombo, Pelotas and, Ponta Grossa) and 78.07 (Fortaleza) (Table 1) and was higher in the north, followed by the northeast, southeast, central-west and south (Table 2). On June 16, 2020, the RDR changed between 0 (Pelotas) and 119.11 (Belém) (Table 1). On July 01, 2020, the RDR changed between 0.28 (Ponta Grossa) and 128.62 (Belém) (Table 1) and was higher in the north, followed by the northeast, southeast, central-west and south (Table 2).

Between May 17 and June 01, between June 02 and June 16, and between June 17 and July 01, the RDR increased respectively 78.4%, 45.5% and 34.6% in Brazil. The largest increase in RDR was observed in the central-west region and the smallest in the north region (Table 2), and the rate of RDR growth decelerated in all regions, except in the southern region (Fig. 2).

Between March 01 and May 17, 2020, the average RH data of 63 cities were analysed, which varied between 52 and 89.4% (Table 1) with an RH average of 74% (Table 2). Between May 18 and June 01, 2020, the average RH data of 58 cities were analysed, which varied between 50.4 and 91.4% (Table 1) with an RH average of 73.8% (Table 2). Between June 02 and June 16, 2020, the average RH of 57 cities varied between 56.1 and 91.9% (Table 1) with an RH of 75.2% (Table 2). Between June 17 and July 01, 2020, the average RH of 58 cities varied between 50.3 and 92.5% (Table 1) with an RH of 70.9% (Table 2). The cities in the central-west region had the lowest average RH values. Cities in the northern and northeast region had the highest RH values (Table 1).

The first confirmed case in Brazil occurred on February 26 of 2020 in the city of São Paulo, and until April 8 of 2020, all cities in Brazil with a population of up to 200 thousand inhabitants confirmed at least one case of COVID-19 (Table 1). There were no significant differences between cities concerning the period of accelerated dissemination. Would, then, the altitude and the RH have contributed to accelerate the spread of the virus in Brazil?

The correlation between altitude and RI calculated from data collected in 154 cities until May 17, 2020, was negative and significant, according to Pearson's correlation (r = − 0.38, n = 154, p < 0.01). The correlation between altitude and RI calculated from data collected in 154 cities until June 01, 2020, was higher (r = − 0.44, n = 154, p < 0.01). The correlation between altitude and RI calculated from data collected in 154 cities until June 16, 2020, was also negative and significant (r = − 0.41, n = 154, p < 0.01). The correlation between altitude and RI calculated from data collected in 154 cities until July 01, 2020, was also negative and significant (r = − 0.36, n = 154, p < 0.01).The same occurred between altitude and RDR until May 17, 2020 (r = − 0.29, n = 154, p < 0.01), until June 01, 2020 (r = − 0.37, n = 154, p < 0.01), until June 16, 2020 (r = − 0.41, n = 154, p < 0.01) and until July 01, 2020 (r = − 0.43, n = 154, p < 0.01).

The correlation between RH and RI calculated from data collected in the period from March 01 to May 17, 2020, in 63 cities (r = 0.48, n = 63, p < 0.01), in the period from May 18 to June 01, 2020, in 58 cities (r = 0.47, n = 58, p < 0.01), in the period from June 02 to June 16, 2020, in 56 cities (r = 0.28, n = 57, p = 0.04), and in the period from June 17 to July 01, 2020, in 54 cities, (r = 0.28, n = 54, p < 0.04) was positive and significant.

The correlation between RH and RDR calculated from data collected in the period from March 01 to May 17, 2020, in 63 cities (r = 0.38, n = 63, p < 0.01), in the period from May 18 to June 01, 2020, in 58 cities (r = 0.37, n = 58, p < 0.01), in the period from June 02 to June 16, 2020, in 56 cities (r = 0.31, n = 56, p = 0.01), and in the period from June 17 to July 01, 2020, in 54 cities (r = 0.30, n = 54, p = 0.02), was positive and significant.

**Figure 3:**
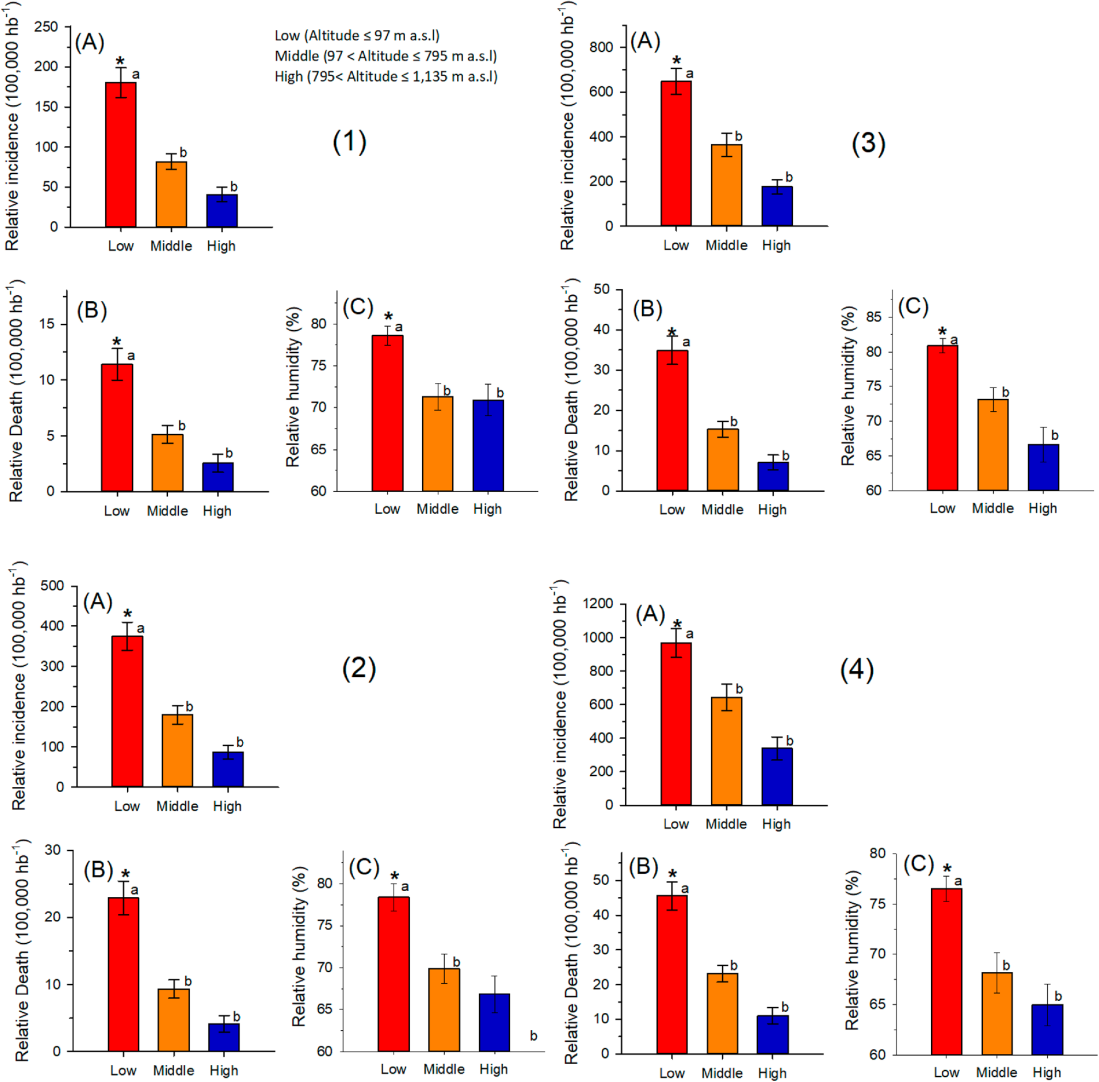
Mean and standard deviation of **(A)** relative incidence (RI); **(B)** relative death rate (RDR) and **(C)** air relative humidity (RH) at low, medium and high altitudes in cities in Brazil with a population above 200 thousand inhabitants (1) until May 17, 2020; (2) until June, 01, 2020; (3) until June, 16, 2020; (4) until July, 01, 2020. *Significant difference by analysis of variance and average values followed by the same letters (superscript a or superscript b) do not differ by the Tukey test (p < 0.05).

The cities were grouped into three classes of altitude: low (altitude ≤ 97 m a. s. l), middle (97 < altitude ≤ 795 m a. s. l), high (795 < altitude ≤ 1135 m a. s. l) (Fig. 2). For RI, RDR and RH, the analysis of variance (hierarchical classification) for data collected until May 17, 2020 (RI: F_2,151_ = 18.80; p < 0.001; RDR: F_2,151_ = 12.46; p < 0.001; RH: F_2,60_ = 7.35; p < 0.001), for data collected until June 01, 2020 (RI: F_2,151_ = 19.40; p < 0.001; RDR: F_2,151_ = 18.64; p < 0.001; RH: F_2,55_ = 7.98; p < 0.001), for data collected until June 16, 2020 (RI: F_2,151_ = 13.40; p < 0.001; RDR: F = 20.35; p < 0.001; RH: F_2,53_ = 10.52; p < 0.001), and for data collected until July 01, 2020 (RI: F_2,151_ = 9.38; p < 0.001; RDR: F_2,151_ = 21,31; p < 0.001; RH: F_2,51_ = 7.16; p = 0.001) was highly significant. The averages of RI, RDR, and RH were compared using the Tukey test, which showed that the incidence is lower at high altitudes in the four analysed periods (Fig. 3).

The r-values of Pearson's correlation between altitude and RI and RDR and the F values of ANOVA for all dependent variables were significant in the four analysed period. Thus, the altitude and the RH may have contributed to accelerate the spread and mortality of the virus in Brazil.

## Discussion

Our epidemiological analysis of the COVID-19 pandemic in Brazil indicates a direct association between the incidence of COVID-19 with altitude and RH in Brazilian cities with a population above 200 thousand inhabitants. The low RI and RDR in cities with higher altitudes may be related to environmental factors, which influence the spread of the virus and the physiology of human beings.

Climatic factors can be determinant for transmission by some viruses^10,11^. Thus, a study performed in Russia assessed possible climatic predictors in the rise of COVID-19 intensity. The results showed that temperature seasonality influenced COVID-19 spread in humid continental Russia, whereas temperature (diurnal range and seasonality) was influential in sub-artic Russia^10^. In addition, another study aimed to examine the linkage between climatic variables and COVID-19 in National Capital Territory (NCT) of Delhi, India. The results of that study suggested that climatic conditions in NCT of Delhi were favourable for COVID-19 and the disease may spread further with the increasing temperature, relative humidity, evaporation and wind speed^11^. However, it is necessary to consider comprehensive biophysical assessments of altitude, humidity, UV radiation in the maintenance and transmission of SARS-CoV-2^12,13^. Recently studies have been published about temperature and UV index, and that heating, and UV radiation can eliminate the viral infectivity^12,13^. These findings could help to understand the relationship of COVID-19 cases in different Brazilian cities due to altitude.

In contrast to what other studies report regarding the greater spread of the influenza virus in environments with lower RH^14^, our data show that COVID-19 RI is higher in cities where RH is highest. These results are in line with those obtained by C Arias-Reyes, N Zubieta-DeUrioste, L Poma-Machicao, F Aliaga-Raudan, F Carvajal-Rodriguez, M Dutschmann, E Schneider-Gasser, G Zubieta-Calleja and J Soliz^3^, which lists air dryness as one of the factors that control the spread of the virus at high altitudes. In working with climatic data from 5 large Brazilian cities (Manaus, Fortaleza, Brasília, Rio de Janeiro, and São Paulo)^15^, showed that moderate relative air humidity (averages between 77.7 and 81.6%) favor the spread of this disease. In the present study, 5 cities with the highest RI, in the four periods analyzed, had an average RH between 79.3 and 84.8%, values similar to those found by A Auler, F Cássaro, V da Silva and L Pires^16^.

Another point that should be considered is that the higher altitude, by itself, favors a higher incidence of ultraviolet (UV) radiation, especially in the UVA and UVB spectra, which can produce a bactericidal effect due to changes in the molecular chains of DNA and RNA. Thus, as a hypothesis, UV radiation would shorten the virus half-life, thereby reducing the virus's ability to survive in Brazilian cities located at higher altitudes and, consequently, the survival of the COVID-19 virus. M Blumthaler, W Ambach, and R Ellinger^17^ observed an increase of 9 ± 2% in UV radiation for every 1000 m of altitude, under clear sky conditions. In addition, the increase in UV radiation can reach 11.3% (5 to 1135 m altitude) between the Brazilian cities studied in this work. Besides, considering that vitamin D production is dependent on exposure to UV radiation and that vitamin D levels positively modulate the immune system^18^, the hypothesis of higher immune defense against SARS-CoV- 2 is plausible in cities with higher altitude. Future studies should investigate this hypothesis.

The low air density and greater distance between molecules in Brazilian cities located at higher altitudes could also reduce the inoculation of airborne viruses compared to sea level. C Arias-Reyes, N Zubieta-DeUrioste, L Poma-Machicao, F Aliaga-Raudan, F Carvajal-Rodriguez, M Dutschmann, E Schneider-Gasser, G Zubieta-Calleja, and J Soliz^3^ suggested that inhabitants of cities with altitudes above 3000 m a.s.l are less susceptible to developing effects caused by COVID-19 due to ultraviolet radiation and thinner air.

Physiological factors can influence the pathogenicity of SARS-CoV-2 at high altitudes. The barometric reading varies with changing weather conditions and becomes lower as the altitude increases. Thus, the volumes of inspired air, which require humidification, are much higher than at sea level, and the air density is lower at high altitudes^19^. As a result, compensatory adjustments to facilitate the release of oxygen to cells occur in individuals living at higher altitudes, such as the increase in the levels of 2,3-diphosphoglycerate (2,3-DPG), a chemical compound found inside the red blood cell, whose function is to reduce hemoglobin's affinity for oxygen in order to facilitate its release into tissues^20^. Given the above, a probable hypothesis for less severity in individuals infected with COVID-19 living in cities with higher altitude could be due to adaptations in these compensatory adjustments to increase the bioavailability of peripheral oxygen. This adaptation is proven in individuals who live at altitudes above 3000 m a.s.l.

**Figure 4:**
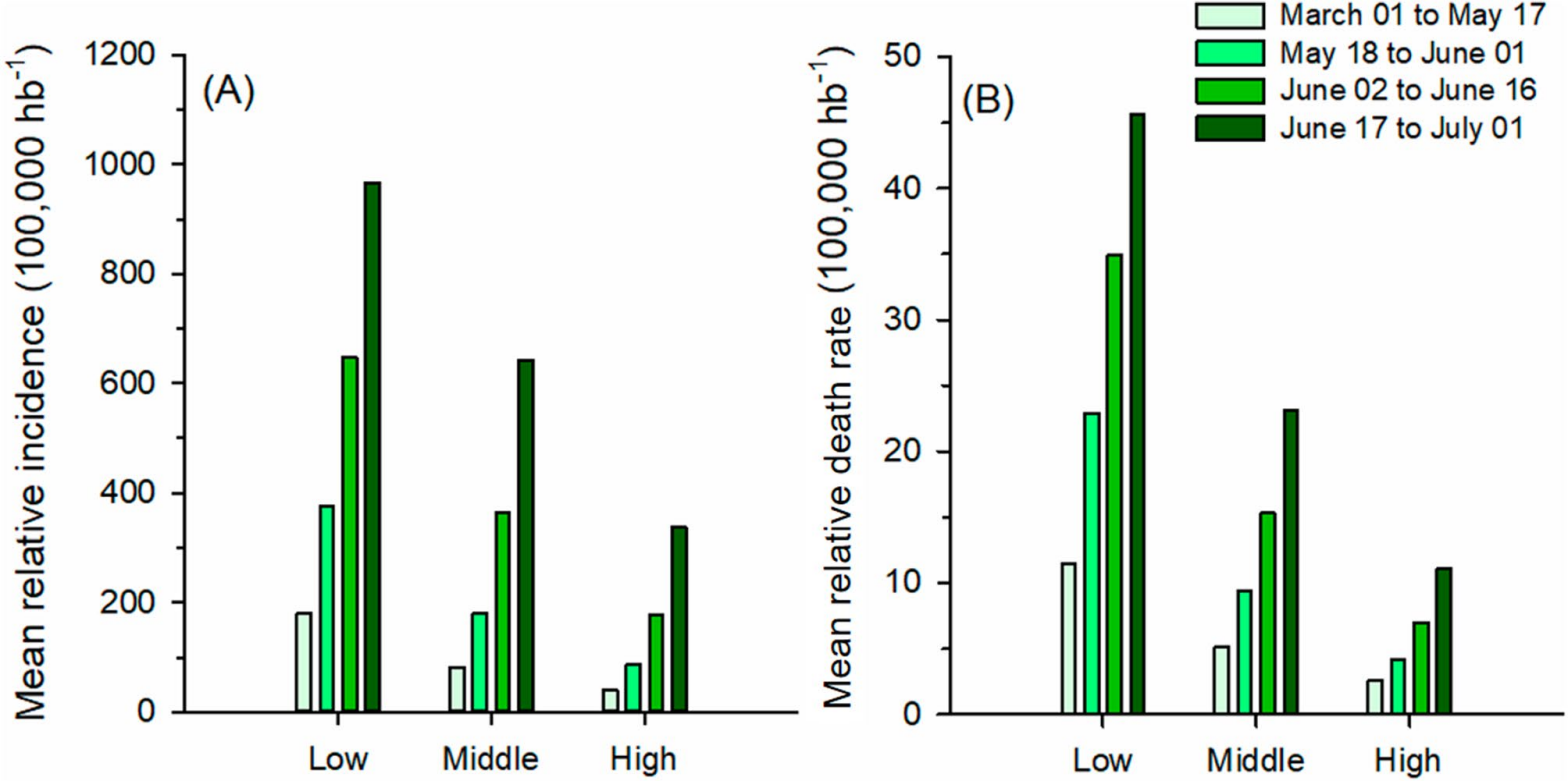
Relative incidence average (RI) and relative death rate average (RDR) in all the cities in Brazil with a population above 200 thousand inhabitants, grouped in three classes of altitude (low, middle and high), for the 4 periods analysed.

**Figure 5:**
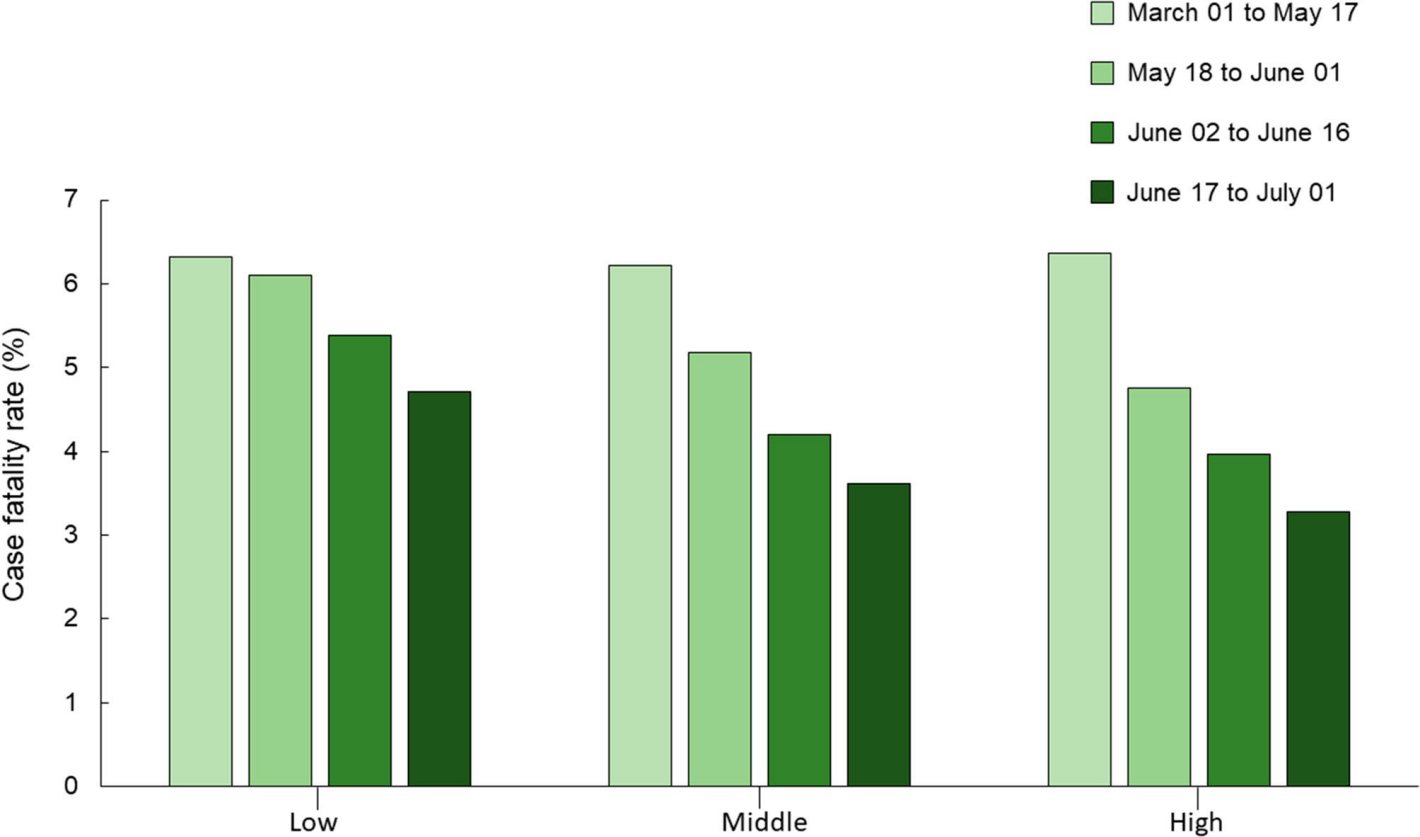
Case fatality rate in all the cities in Brazil with a population above 200 thousand inhabitants, grouped in three classes of altitude (low, middle and high), for the 4 periods analysed.

Other studies have also investigated the relationship between altitude, infection, and case fatality by COVID-19. In Peru, infection by COVID-19 reduced with increasing altitude. However, case-fatality rate was not dependent of altitude^20^. In addition, case studies in the USA and Mexico showed that mortality due to COVID-19 was greater in cities with altitude higher than 2000 m versus located lower than 1500 m^21^. A publication from Italy showed no association of COVID-19 with altitude^22^. In our study, RI and RDR became lower as the altitude increase (Fig. 4). In addition, although the case fatality rate (deaths/cases) decreased over the period analysed for all altitude classes, the reduction was greater at high altitudes (Fig. 5). In this way, such adaptations, even to a lesser extent, could contribute to minimizing the severity of infection in cities located at higher altitudes.

The casual movement of dissolved oxygen molecules establishes the PO_2_ of plasma and tissue fluids. The pressure of oxygen in the solution helps to regulate breathing, particularly at higher altitudes, when the ambient PO_2_ decreases considerably; it also determines the uptake of oxygen by haemoglobin in the lungs and the subsequent release into the tissues. However, haemoglobin saturation with oxygen changes very little until the oxygen pressure has decreased to about 60 mmHg. Even when alveolar PO2 drops to 75 mmHg, as it does at high altitudes, haemoglobin saturation decreases by only approximately 6%. At 60 mmHg alveolar PO2, haemoglobin is still 90% saturated with oxygen. Below that pressure, the volume of oxygen combined with haemoglobin decreases more quickly.

As exposed, the S shape of the oxyhaemoglobin dissociation curve indicates that there is only a small change in the percentage saturation of haemoglobin with oxygen up to an altitude of approximately 3,048 m. At 1,981 m, for example, the alveolar PO2 falls from its value at sea level from 100 to 78 mmHg^3^. However, haemoglobin remains 90% saturated with oxygen.

In addition, regarding the possible physiological factors, the receptor-binding domain (RBD) in the SARS-CoV-2 protein was recently identified and that the RBD protein-bound firmly to the receptors of the human angiotensin-converting enzyme 2 (ACE2)^23^. Human ACE2 is part of the renin-angiotensin system (RAS), an essential hormonal system for controlling blood pressure and fluid and electrolyte balance. In the classical view, RAS peptides are generated from a single precursor protein called angiotensinogen (ATG). After being cleaved by the protease renin, this protein forms the inactive decapeptide angiotensin I, which is hydrolysed by the angiotensin-converting enzyme (ACE) and forms the octapeptide angiotensin II (Ang II), the principal peptide in the system. ACE2 cleaves a single residue of angiotensin I (Ang I) that generates the Ang- (1–9) peptide and degrades Ang II to the Ang- (1–7) vasodilator. Current data obtained during the pandemic suggest that the use of ACE inhibitors and angiotensin type I receptor blockers increase the expression of ACE2. Consequently, increased expression of ACE2 would facilitate infection by COVID-19^24,25^. Studies show that RAS elements are modulated at high altitudes^26–28^. In this way, it is possible that the expression of ACE2 can be down-regulated due to the high altitude and favour a lower incidence of COVID-19 infection. Other studies speculate in this direction and suggest different paths for an ACE2 down-regulation^3,29^, but future studies need to clarify this hypothesis.

Given the above, considering that the Brazilian cities studied at a higher altitude are around 1100 m^5^, probably the hypothesis of compensatory adjustments related to ACE2 in pulmonary epithelial cells, i.e., a protective factor for virus penetration and evolution of severe pulmonary edema, should be studied with caution in residents of Brazilian cities with higher altitudes. Furthermore, this population is also not exposed to conditions of chronic hypoxia. The results presented in this work can be useful for the implementation of public policies for prevention, control of the dissemination of COVID-19 in Brazil and the world. In addition, it can contribute to future studies, including other zoonotic viruses that cause respiratory diseases, as well as allowing the recommendation of changing the environment for people at risk in COVID-19.

Our findings identified that virulence by SARS-CoV-2 is lower in Brazilian cities with a population above 200 thousand inhabitants, located at relative high altitudes, and where the RH is lowest (Fig. 3). These findings are in line with the physiological compensatory adjustments of the inhabitants of cities located at higher altitudes, as well as with the common characteristics. Thus, our study starts point for future studies to establish causality of environmental conditions with SARS-CoV2, contributing to the implementation of measures to prevent and control the spread of COVID-19. It is, however, important to note that the information presented here clearly lacks any physiological evidences, which may merit further investigation. In addition, we cannot assume uniform density population in every city studied, as well quality of care, probability of infection, among other ecological variables, that may affect the outcome. In other words, future studies should consider those variables. As prospective, longitudinal studies are needed to confirm whether these associations remain over time.

## Data Availability

The datasets used and/or analysed during the current study are available from the corresponding author on reasonable request.
